# Editorial

**DOI:** 10.1038/s43705-021-00013-3

**Published:** 2021-04-14

**Authors:** Janet E. Hill, Jos M. Raaijmakers, Hauke Smidt

**Affiliations:** 1 Department of Veterinary Microbiology, Western College of Veterinary Medicine, University of Saskatchewan, Saskatoon, Canada; 2 Department of Microbial Ecology, Netherlands Institute of Ecology, Wageningen, The Netherlands; 3 Laboratory of Microbiology, Wageningen University & Research, Wageningen, The Netherlands

**Keywords:** Ecology, Microbial ecology

The moment has come! We are truly excited to welcome you to *ISME Communications*, a new online, open access journal to publish the latest advances in Microbial Ecology. After various discussions with peers, we proudly present here the first issue of this new journal, supported by ISME and brought to you by Springer Nature.

You may wonder, why we need a second journal in addition to *The ISME Journal* that has been THE PLACE TO BE for microbial ecological research? More importantly, ‘What will *ISME Communications* offer and mean to the scientific community?’

Microbial ecologists around the globe study the stunning microbial diversity that originates from the endless number of ecological niches available in natural and man-made environments. Due to evolutionary processes, microorganisms have adapted to virtually all conditions on earth and have shown an amazing repertoire of functional traits, many of which are yet to be discovered. Microbial communities play crucial roles in local and global elemental cycles on our planet; they determine soil fertility at a local scale, but also drive and react to environmental perturbations acting at a global scale like climate change.

*The ISME Journal* has, for almost 14 years, provided a central platform for the promotion of diverse and integrated areas of microbial ecology spanning the entire breadth of microbial life, including bacteria, archaea, eukaryotes, and viruses/phages. *The ISME Journal*’s strength continues to lie in its focus on contributions that are of broad ecological interest and impact. A recent survey among the growing community of ISME members pointed to the genuine need for more space to publish other top-quality manuscripts that cannot be accommodated by *The ISME Journal*. Particularly, members overwhelmingly advocated for an additional journal to publish microbial ecology research that is of a different scope and impact but highly complementary to that of *The ISME Journal*. With that in mind, it was only a small step to initiate a second journal supported by ISME—*ISME Communications* was born. This “birthing” process would not have been possible without the strong support from the ISME Executive Board, Sarash de Wilde (ISME office), and the Editors-in-Chief of *The ISME Journal*. Particularly, we are indebted to Prof. Mark Bailey, who has been the main initiator of *ISME Communications* from the very beginning.

*ISME Communications* will focus on methodological and computational studies that represent major advances for the field of microbial ecology and also on high-quality, discovery-based and observational research covering topics that are at the heart of microbial ecologists around the globe:

- Climate change microbiology.
- Community assembly.
- Engineered and synthetic microbial communities.
- Host-microbiome interactions.
- Methodological and computational advances.
- Microbiomes of engineered environments.
- Modelling and ecological theory.
- Novel microorganisms and metabolic functions.
- Spatial and temporal dynamics.

It is our mission to make *ISME Communications* as successful as its sister journal. To this end, its exclusively online publication format enables us to publish articles as soon as they are ready, thus benefiting authors by allowing earlier publication and giving readers access to accepted papers weeks before they would appear in a print issue. Together with *The ISME Journal*, *ISME Communications* will now provide all of us with an extended platform for publishing our top-level research, contributing to major progress and impact of the field. In order to provide our readers with valuable insight into the newest and emerging developments in the field of microbial ecology, upcoming issues will feature a series of mini-reviews on particularly new developments in this exciting field.

